# A Global Clustering Algorithm to Identify Long Intergenic Non-Coding RNA - with Applications in Mouse Macrophages

**DOI:** 10.1371/journal.pone.0024051

**Published:** 2011-09-30

**Authors:** Lana X. Garmire, David G. Garmire, Wendy Huang, Joyee Yao, Christopher K. Glass, Shankar Subramaniam

**Affiliations:** 1 Department of Bioengineering, Jacobs School of Engineering, University of California San Diego, La Jolla, California, United States of America; 2 Department of Electrical Engineering, University of Hawaii at Manoa, Honolulu, Hawaii, United States of America; 3 Department of Cellular and Molecular Medicine, School of Medicine, University of California San Diego, La Jolla, California, United States of America; Georgia Institute of Technology, United States of America

## Abstract

Identification of diffuse signals from the chromatin immunoprecipitation and high-throughput massively parallel sequencing (ChIP-Seq) technology poses significant computational challenges, and there are few methods currently available. We present a novel global clustering approach to enrich diffuse CHIP-Seq signals of RNA polymerase II and histone 3 lysine 4 trimethylation (H3K4Me3) and apply it to identify putative long intergenic non-coding RNAs (lincRNAs) in macrophage cells. Our global clustering method compares favorably to the local clustering method SICER that was also designed to identify diffuse CHIP-Seq signals. The validity of the algorithm is confirmed at several levels. First, 8 out of a total of 11 selected putative lincRNA regions in primary macrophages respond to lipopolysaccharides (LPS) treatment as predicted by our computational method. Second, the genes nearest to lincRNAs are enriched with biological functions related to metabolic processes under resting conditions but with developmental and immune-related functions under LPS treatment. Third, the putative lincRNAs have conserved promoters, modestly conserved exons, and expected secondary structures by prediction. Last, they are enriched with motifs of transcription factors such as PU.1 and AP.1, previously shown to be important lineage determining factors in macrophages, and 83% of them overlap with distal enhancers markers. In summary, GCLS based on RNA polymerase II and H3K4Me3 CHIP-Seq method can effectively detect putative lincRNAs that exhibit expected characteristics, as exemplified by macrophages in the study.

## Introduction

Unlike messenger RNA, non-coding RNAs (ncRNAs) are a class of RNAs that are not intermediates between DNA and protein products. Rather than being regarded as “transcriptional noise”, there is emerging recognition and appreciation of the functional importance of these ncRNAs in health and diseases, such as cancer [1]. According to the length of transcripts, ncRNAs can be classified into three categories: small RNA (≤25 bp), medium-length RNA (∼30–200 bp), and long RNA (longer than 200 bp) [2]. The understanding of ncRNA biology is evolving rapidly as more and more ncRNAs are being discovered. For example, it was previously thought that ncRNAs lacked evolutionary conservation; however, recent studies revealed compelling evidence supporting the conservation of lincRNAs [3], [4]. In addition, there is emerging evidence that lincRNAs play roles in regulation of gene expression, in part through targeting transcriptional complexes to specific genomic locations [5], [6].

RNA polymerase II (Pol II) plays a central role in transcribing both coding and non-coding RNAs. The ability of Pol II to initiate transcription is dependent upon the combinatorial functions of general and sequence–specific transcription factors that establish an open chromatin template and mark the site of transcriptional initiation. This process is dependent on nucleosome remodeling factors and histone modifying enzymes that mark histone tails with specific post-translational modifications serving as docking sites for transcriptional co-regulatory proteins. Trimethylation of histone H3 lysine 4 (H3K4me3) is a well-known marker on promoter regions correlated to gene activation, and trimethylation of histone H3 lysine 36 (H3K36me3) is another marker along a given transcribed region [3]. Thus, an RNA transcript can likely be identified by finger printing the positions of a chain of Pol IIs that are closely adjacent to each other. H3K4me3 signatures within such boundaries provide additional information to validate the active transcription status.

High resolution snap shots of active transcription are recently enabled by chromatin immunoprecipitation (ChIP) combined with ultra high-throughput massively parallel sequencing technologies, also known as ChIP-Seq technology [7]. ChIP assay pulls down the genomic DNA segments where proteins of specific interest are bound and ultra high-throughput massively parallel sequencing technology provides digitized readouts of these DNA segments.

The CHIP-Seq signatures of DNA associated proteins such as of Pol II and histone markers are diffuse and span a wide range from several nucleosomes to thousands of bps. Such signals are not easily detected by existing peak-finding algorithms for transcription factor binding, such as FindPeaks [8]. In fact, most current algorithms have been designed for analyzing sharp peaks from the transcription factors, but not the diffuse CHIP-Seq signals. One exception is the study of Guttman et. al, in which they developed a method to adjoin neighboring diffuse histone modification signatures of H3K4Me3 and H3K36Me3, in order to discover long intergenic noncoding RNAs (lincRNAs) [3]. They defined the putative lincRNAs as the maximum continuous sequences over multiple windows that have tag counts larger than a predefined number of tag counts, and have significant P-values based on a Poisson null model. Zang et al. developed SICER, a clustering method to find islands of signatures in diffuse signals. In their method, gaps are allowed to merge neighboring signals with high island-scores. False discovery rate (FDR) is then used to keep the regions that have high scores over a randomized back-ground model or input data [9]. Here, we report a global clustering approach that identifies correlated diffuse CHIP-Seq peak signals likely to belong to the same unit based on the distribution of peak width and inter-peak distance. We run simulations showing the observed correlation appears to arise from reaction-diffusion dynamics. We apply this method to Pol II and H3K4Me3 CHIP-Seq data to find lincRNAs, and compare its performance to SICER, a publicly available computational method to merge diffuse CHIP-Seq signals.

The data in this study are obtained from primary macrophage cells under resting and activated conditions. Deriving from circulating monocytes in the blood stream, macrophages are terminally differentiated cells with important innate and adaptive immune functions [10]. In addition, they contribute to different pathological conditions, such as arthrosclerosis, diabetes, cancers and various autoimmune diseases [10], [11], [12], [13]. Lipopolysaccharides (LPS), a component of the outer membrane of Gram-negative bacteria that activates toll-like receptor 4 (TLR4), can massively and rapidly change the macrophage gene expression program, including induction of an acute inflammatory response that results in the production of large quantities of cytokines and chemokines [14], [15]. Resting and activated macrophages therefore provide a powerful model system for identification of lincRNAs that are expressed under basal conditions as well as those that are regulated by inflammatory stimuli [16].

## Results

### Emerging pattern of diffuse CHIP-Seq peaks

**Figure 1:**
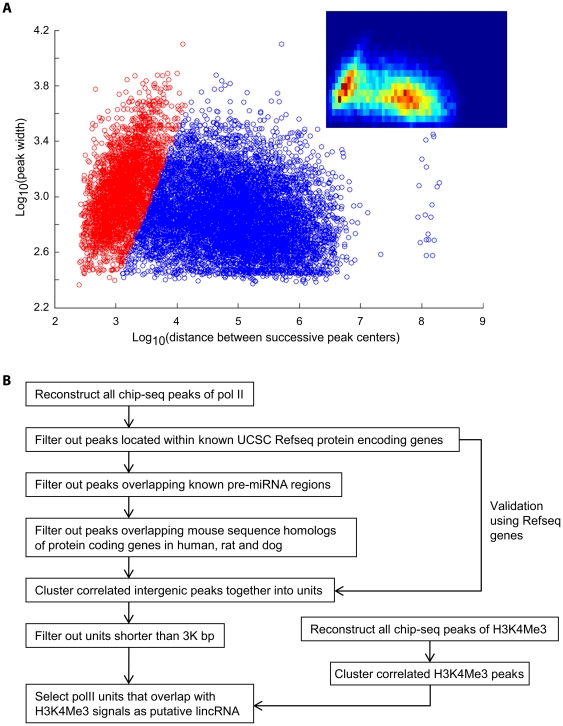
genome-wide patterns of diffuse CHIP-Seq peaks and their application to lincRNA discovery. A: the emerging patterns of Pol II CHIP-Seq peaks. Data are displayed as log10 transformation of the width of peaks vs. log10 transformation of the distance between two successive peak centers. Red data points denote peaks that appear to be linearly correlated between peak width and inter-peak distance. Blue data points denote peaks that lack the linear correlation between peak width and inter-peak distance. The linear separator that separates the two types of peaks is determined by iterative computation described in Methods. Insert: the density heat-map plot of data points in Figure 1. B: flowchart to demonstrate the process of filtering and clustering Pol II and H3K4me3 peaks to identify lincRNAs.

Unlike the local-clustering approach of Zang et. al [9], we applied a global approach to explore the patterns of diffuse CHIP-Seq peaks. We used Pol II CHIP-Seq data as examples, but the pattern holds in general for histone marker data such as H3K4me3, H3K4me1 and H3K27me3 (data not shown). From the density plot of the logarithm-transformed genomic peak width versus logarithm transformed distance between the nearest genomic peaks (Figure 1A), we discovered two groups of peaks. One group had little correlation between peak width and interpeak distance (blue). The other group had a linear-like correlation between peak width and interpeak distance (red). This pattern is maintained when we separately plotted the peaks that are located within RefSeq genes, and the peaks that reside intergenically (Figure S1). Interestingly, the K-mean (K = 2) clustering method with the cosine metric also generated two clusters separated by a line (Figure S2). These results indicated that there are two types of peak distributions – one type (Type 1) that represent a group of peaks that were close together and likely a part of a larger lincRNA unit, and another type (Type 2) that are more likely unrelated peaks. We observed a linear relationship for the Type 1 peaks, which indicated that as these peaks became wider, the separation between peaks became larger. Conceptually, one can rationalize that the Type 1 peaks reflect the dynamics of “diffusion-reaction”, an emergent picture of limited amount of enzymes competing for specific genomic loci, assuming that the enzymes are diffused to targeted loci and that the total loading capacity of the enzymes within a particular actively transcribed locus is relatively constant. Due to the stochastic nature, the more likely one sees some enzymes aggregating closely as one single, wide peak, the farther apart one would expect to see the other competing enzymes near that particular locus.

Motivated by this observation, we constructed a simplified simulation of the Pol II signals modeled over time assuming a simple one-dimensional reaction-diffusion system, in order to demonstrate that the above hypothesis is plausible. . We assume the distribution of Pol II signal to be uniform and the diffusion of the signal followed Fick's second law with a selected diffusion coefficient (*D*). We carried out several computations in each step, including determining the potential binding sites, signal amplification upon binding, and calculating the simulated Pol II expression levels. We used the first 300 Mbps of Chromosome 1 for the representative gene data and show a resulting distribution after a time evolution of these signal peaks in Figure S3. This distribution is similar to what was found in the observed CHIP-Seq data, indicating that the reaction-diffusion dynamics may be responsible for generating the observed distribution. The description of the relevant parameters of the simulation is detailed in the Methods.

Based on this two-cluster pattern, we implemented an iterative global-clustering-over-linear-separator (GCLS) algorithm to reconstruct the most correct transcription units from these peak components (see Methods). Briefly, Type 1 peaks were defined as the remaining peaks on one side of the best linear separator going through the point of minimum density between the two groups. Then the Type 1 peaks were merged with their nearest neighbors. A new density plot was made after such merging, to calculate a new best linear separator. This process was iterated until convergence. H3K4Me3 signatures were clustered similarly. We define a putative lincRNA transcript as the Pol II clusters that overlap with at least one H3K4Me3 cluster. The flowchart of finding lincRNA is shown in Figure 1B, following the similar filtering scheme as others [3]. We used slightly different minimum length threshold of 3 Kbp for the lincRNAs (see Methods).

### Evaluation of GCLS method using known protein-coding genes as test sets

**Figure 2:**
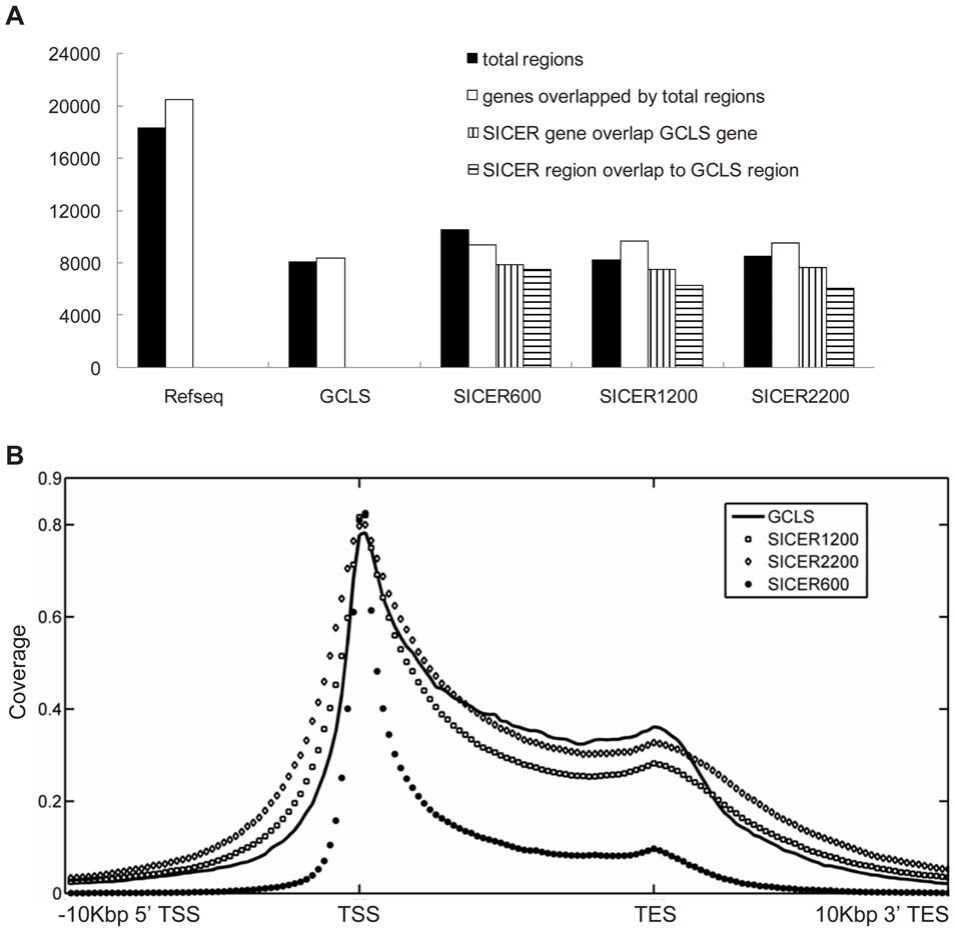
Comparison of GCLS and SICER on RefSeq genes. A: comparison of the performance of SICER against GCLS among RefSeq genes. SICER is parameterized over 3 different gap distances: 600 bp, 1200 bp and 2200 bp. A region is defined as the maximum contig of several overlapping genes if there are any, or the locus of one single gene if there are no other overlapping genes. The total RefSeq regions and genes are plotted as the references to illustrate the fraction of regions and genes that are actively expressed in macrophages. B: comparison of the coverage of GCLS vs. SICER on RefSeq genes spanning from 10 kbp upstream of the transcription start site (TSS) to 10 kbp downstream of the transcription end sites (TES). The transcription regions (TSS-TES region) of genes of different length are normalized to the same effective length. This region was subdivided into 50 bins and the coverage was counted in each bin. Similarly, the 10 kbp region upstream of TSS and the 10 kbp region downstream of TES were also subdivided into 50 bins and counted the coverage in each bin. GCLS has the best coverage in the transcript region, and second lowest noise level in the upstream of TSSs and the downstream of TES.

Before applying the GCLS method to discover lincRNAs, we first tested it on Pol II peaks located within RefSeq genes, and compared the result to that of the comparison method SICER, in three different gap parameter settings: 600 bp (SICER600), 1200 bp (SICER1200), and 2200 bp (SICER2200). We show the summary of comparison in Figure 2A. Among the over 18,000 RefSeq regions (overlapping genes are concatenated as one region), GCLS identifies over 8100 transcriptional regions. By comparison, SICER with various parameters identify similar but slightly more regions. However, GCLS has the least discrepancy between the total number of identified transcriptional regions and the actual RefSeq gene counts, compared to the results of SICER. In fact, 64% of the predicted regions correspond to single genes in GCLS, compared to 63.5% (P = 0.385) in SICER600, 50% (P = 1.67e-08) in SICER1200 and 55% (P = 0.96e-21) in SICER2200. SICER600 produced more regions than actual gene counts. On the other hand, SICER1200 and SICER2200 allow too large gap distances that a region found in these conditions can harbor multiple genes. Nevertheless, there are a large percentage of genes and regions identified by SICER that overlapped the genes and regions identified by our GCLS method (Figure 2A), and vice versa (Figure S4). The Receiver-Operator Curve (ROC) plot based on the binary classification of known genes identified by Pol II vs. microarray gene expression prediction also shows that, GCLS and SICER can extract clusters that overlap with known genes with very similar trends of true positive rate vs. false positive rate (Figure S5).

To further evaluate GCLS vs. SICER on the ORF of RefSeq genes, we investigated the coverage of these two methods in the 5′-upstream of TSS (transcription starting site), TSS-TES (transcription ending site) and 3′-downstream of TES from the identified genes (Figure 2B). Interestingly, Pol II coverage presents a bi-modal distribution under either method, indicating Pol II stalling near the promoter regions and the TES. The stalling phenomenon was reported earlier near the promoters [17], [18], [19], [20], [21], but minor stalling near TES has not been shown previously according to our knowledge. GCLS method and SICER2200 have very similar coverage profiles in the coding region, better than SICER1200 and much better than SICER600. GCLS performs even better than SICER2200 near the TESs. Overall GCLS covers about 80% of TSS and gradually decreases to about 40% near TESs. In the 5′ upstream of TSSs and 3′ downstream of TESs, the noise levels present in the order of SICER600<GCLS<SICER1200<SICER2200. In summary, GCLS is better than SICER2200 and SICER1200 in both accuracy and coverage. GCLS is much superior to SICER600 in terms of the coverage in coding regions, but not as accurate beyond the ORF. However, due to the much larger distance between intergenic peaks (Figure S1), SICER600 is expected to perform much more poorly than GCLS, because SICER600 is dependent on the gap-distance whereas GCLS is independent of it. Due to this reason, as well as the least discrepancy between regions and gene counts shown in Figure 2A, we chose to rely on our global clustering approach for the lincRNA discovery.

### lincRNA prediction with GCLS

**Figure 3:**
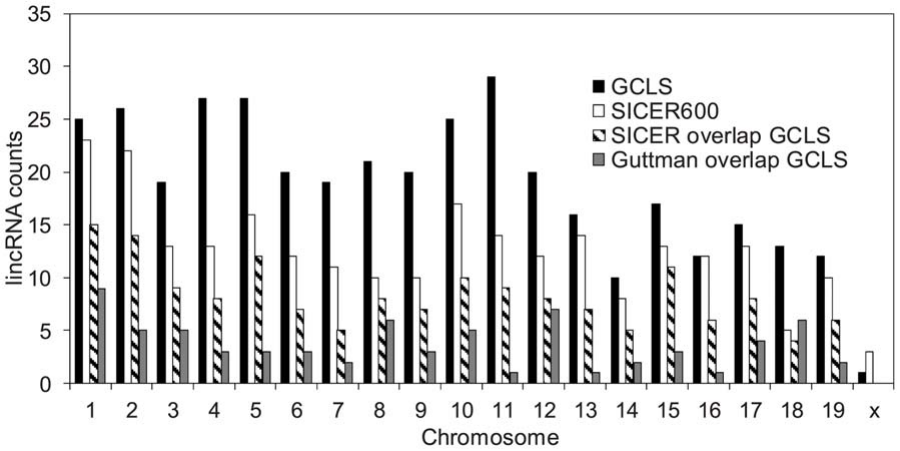
Comparison of the lincRNA overlap among three different methods: GCLS, SICER600 and Guttman's method in [**3**]. The counts of lincRNAs are grouped in the ascending order of chromosomes.

We found a total of 374 putative lincRNAs in macrophages under the no treatment condition and 189 lincRNAs under LPS treatment (Table S1). We compared the results of GCLS from the no treatment condition to those of SICER600. Overall GCLS compares favorably to SICER600. As expected, GCLS found more putative lincRNAs than SICER600 (251 units). Moreover, GCLS was capable of recovering putative lincRNAs of larger average lengths and length variations. The lincRNA units from GCLS have an average length of 12,474 bp with a standard deviation of 13,703 bp, whereas the units from SICER have an average length of 5215 bp with a standard deviation of 2363 bp. This indicates that GCLS is more flexible at predicting putative lincRNAs of a broader range of lengths, comparing to SICER, which predefines the inter-island gap distance. A majority of the peaks found through GCLS overlapped with those from SICER (Figure 3, Table S2). Of the 251 units found by SICER, 159 or 63% overlapped with these found by GCLS. These lincRNA units from macrophage did not appear to have large overlap with those reported by Guttman et al. [3], which were found in mouse embryonic stem cells (ESCs), mouse embryonic fibroblasts (MEF), mouse lung fibroblasts (MLF) and neural precursor cells (NPC) (Table S2).

### Effect of LPS on lincRNAs and experimental validation

**Figure 4:**
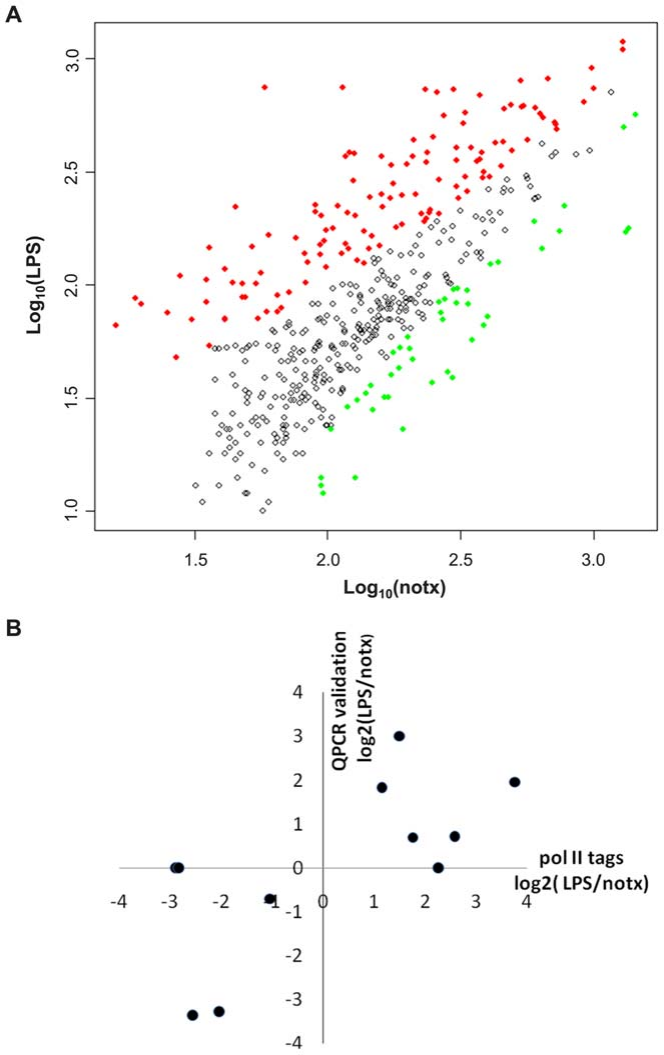
Effect of LPS on lincRNAs. A: differentially regulated lincRNAs by LPS. Due to the difference in tags between the LPS vs. no-treatment conditions, tag counts under the no-treatment condition are first normalized by a linear regression, and then tested for difference as described in Methods. Data plotted are the log10 transformation of the original tag counts in LPS treatment vs. the log10 transformation of the original tag counts in LPS treatment. Red data points (126) denote up-regulation and green data points (45) denote down-regulation. B: experimental validation of 11 lincRNAs. Data are plotted as log2 transformation of fold change on predicted exons by QPCR experiments vs. log2 transformation of fold change in Pol II tag counts by QPCR experiments. The lincRNAs that are under-detectable in QPCR are assigned to have y-values of 0.

As Toll-like receptor (TLR) activation dramatically alters the transcription program of protein-coding genes in macrophages [22], [23], we hypothesize that transcription of lincRNAs may also be TLR responsive. Towards this goal, we tested the Pol II tag differential distribution in the same putative lincRNA regions from LPS treatment vs. no treatment. We first normalized the tag difference between LPS treatment vs. no treatment (see Methods). We then utilized a binomial model to perform the statistical test. We detected 45 putative lincRNAs with significantly fewer Pol II tags, and 126 putative lincRNAs with significantly more Pol II tags under LPS treatment vs. no treatment (Figure 4A).

We selected 11 regions from the putative lincRNAs for experimental validation (Table 1). Within these regions, 6 have increased Pol II tag counts upon the LPS treatment, 4 have reduced Pol II tag counts by the LPS treatment and one did not change significantly. LincRNAs have features of exons [3], [4], [24], and hence we predicted exons (posterior P-value>0.10 as suggested in the software. For convenience all the probabilities in the paper are posterior probabilities, unless noted otherwise) using GENSCAN software and designed primers spanning the exons (100–200 bp) in each region. QPCR validation was performed on RNAs extracted from bone marrow derived primary macrophages under no treatment, or LPS treatment conditions. These experiments showed that tested putative lincRNAs had low to medium expression levels, compared to GAPDH (Figure S6). 5/6 putative lincRNAs were indeed induced by LPS (except chr11, whose expression was below detection threshold), and 2/4 putative lincRNAs were indeed reduced by LPS (except chr3 and chr5, whose expressions were very low). The fold changes of QPCR upon the LPS treatment to those of tag counts of Pol II ChIP-Seq is presented in Figure 4B. The correlation coefficient is as high as 0.86 for the 8 putative lincRNAs that were expressed.

**Table 1:** Experimental validation on 11 regions of lincRNAs.

chrom	segment start	segment end	strand	Pol II status	Primary Macrophage Validation
chr13	55198800	55202000	+	LPS_induced	Y
chr11	83349000	83361500	+	LPS_induced	no-detect
chr2	30734000	30755000	−	LPS_induced	Y
chr10	18750000	18758500	+	LPS_induced	Y
chr9	119857500	119863000	+	LPS_induced	Y
chr17	29119500	29124500	−	LPS_induced	Y
chr3	84730000	84735000	−	LPS_reduced	Y, low
chr6	48950000	48964000	−	LPS_reduced	Y
chr14	62032000	62036800	+	LPS_reduced	Y
chr5	37283500	37290000	+	LPS_reduced	Y, low
chr8	87090000	87103500	+	no_change	Y

### Computational Characterization of lincRNAs

The correlation between lincRNA Pol II tag fold change upon LPS and its nearest RefSeq gene expression fold change upon LPS treatment was reasonably good (Pearson's correlation coefficient = 0.44, P-value = 2.145624e-26). Based on the idea that lincRNA would most likely regulate their nearest genes [3], we associated the lincRNAs with their nearest RefSeq genes, in order to predict the biological function of these putative lincRNAs. We performed GO analysis on biological processes with a cut-off threshold of FDR<0.01. LPS treatment and no treatment both yield primary metabolic process and regulation of cellular process (Table 2). lincRNAs expressed in the basal condition were near genes enriched for metabolic processes. LPS treatment shifts the genes near lincRNAs towards immune cell specific processes such as lymphocyte differentiation. Similarly, KEGG pathway analysis (P-value<0.01) also shows that LPS treatment shifts lincRNA program to immune response related pathways such as cytokine-cytokine receptor interaction, as expected (Table 2).

**Table 2:** GO (top) and KEGG pathway (bottom) analysis of RefSeq genes that are nearest lincRNAs.

experiment	GO term	Genes	FDR%
T	lymphocyte differentiation	7	0.5
	immune system process	19	0.6
	B cell differentiation	5	0.9
T|U	primary metabolic process	149|81	0|0.7
	regulation of cellular process	91|51	0|0.6
U	intracellular singaling cascade	40	0
	biological regulation	105	0
	regulation of biological process	97	0.1
	macromolecule metabolic process	131	0.1
	protein kinase cascade	16	0.1
	cellular metabolic process	143	0.5
	metabolic process	156	0.5
	MAPKKK cascade	10	0.7

experiment	KEGG pathway	Genes	P-value
T	cytokine-cytokine receptor interaction	9	5.00E-03
	MAPK signaling pathway	9	6.40E-03
	VEGF signaling pathway	5	7.90E-03
U	leukocyte transendothelial migration	10	1.80E-04

T: treated with LPS; U: control condition untreated with LPS.

**Figure 5:**
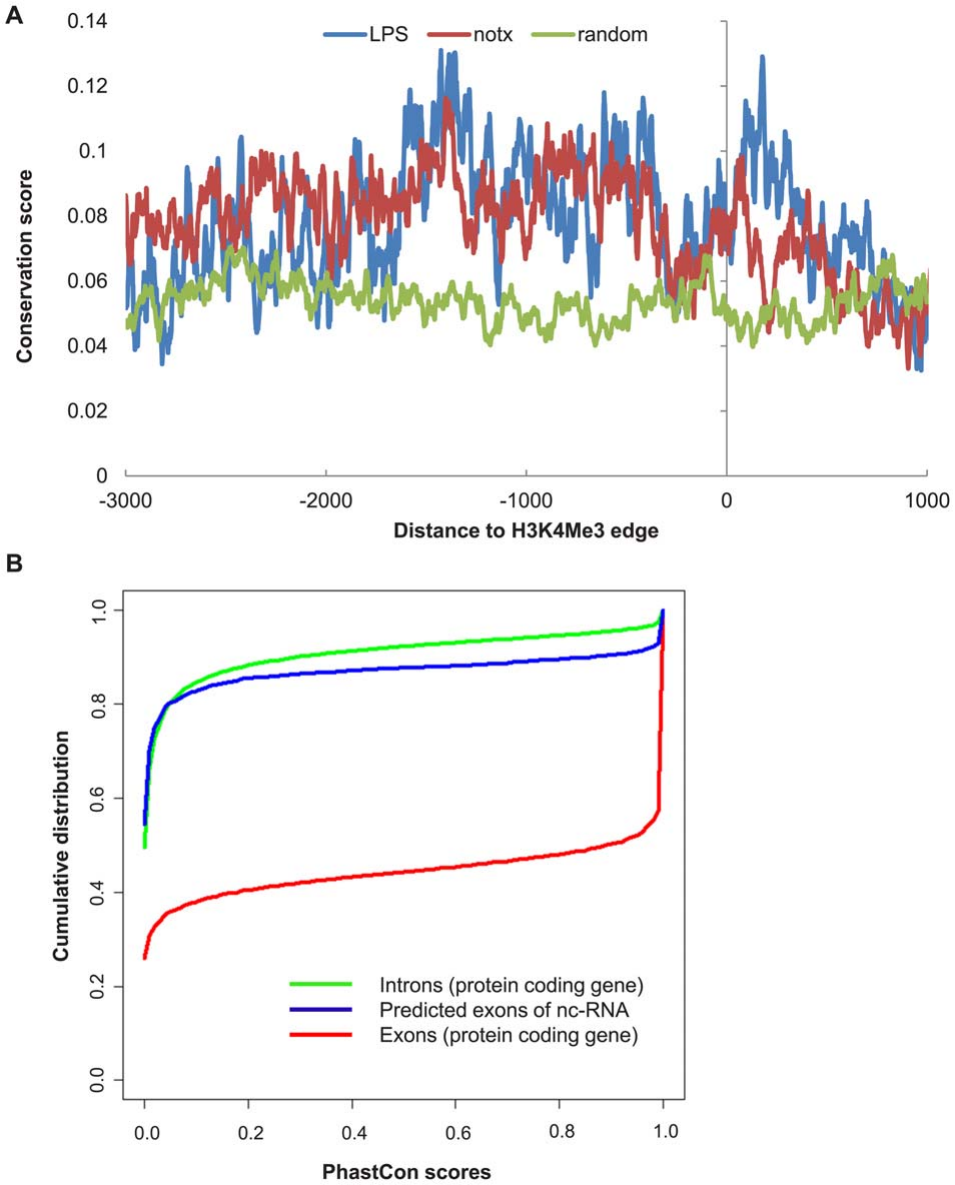
Conservation in lincRNA. A: conservation in the promoter region of lincRNA. The promoter region is defined as −3 K to 1 K relative to TSS that is labeled by the 3′ edge of the H3K4Me3 peaks. Averaged phastCon scores are used as measurements of conservation. Random intergenic sequences without evidence of lincRNAs are plotted as the control. Both LPS and no-treatment have significant higher phastCon scores than the random sequence (Wilcoxon tests, P-value<1e-15). B: conservation of predicted exons of lincRNA, in comparison to the introns and exons of protein coding genes. The accumulative fractions of phastCon scores are plotted against the phastCons. The predicted exons of lincRNAs are modestly conserved compared to the introns of protein coding genes, but are much less conserved compared to the exons of protein coding genes.

To investigate the evolutionary conservation among the putative lincRNAs, we carried out analysis in the predicted exons by GENSCAN as well as their respective predicted promoter regions labeled by H3K4Me3 signatures. By comparing the cumulative distribution of PhastCon scores of the predicted exons of the lincRNA to those of the introns and exons of protein coding genes, it is clear that overall the predicted exons of lincRNAs are slightly more conserved than the collective introns of the protein coding genes on average. They are, however, much less conserved than the exons of the protein coding genes (Fig. 5B). This result is consistent with earlier observations [3], [4], [24]. Furthermore, the promoters of the putative lincRNAs also show evidence of conservation (Fig. 5A). H3K4Me3 signatures have been regarded enriched in the promoter region. Correspondingly, the conservation scores are significantly higher in this region (Wilcoxon tests P-value<1e-15).

**Figure 6:**
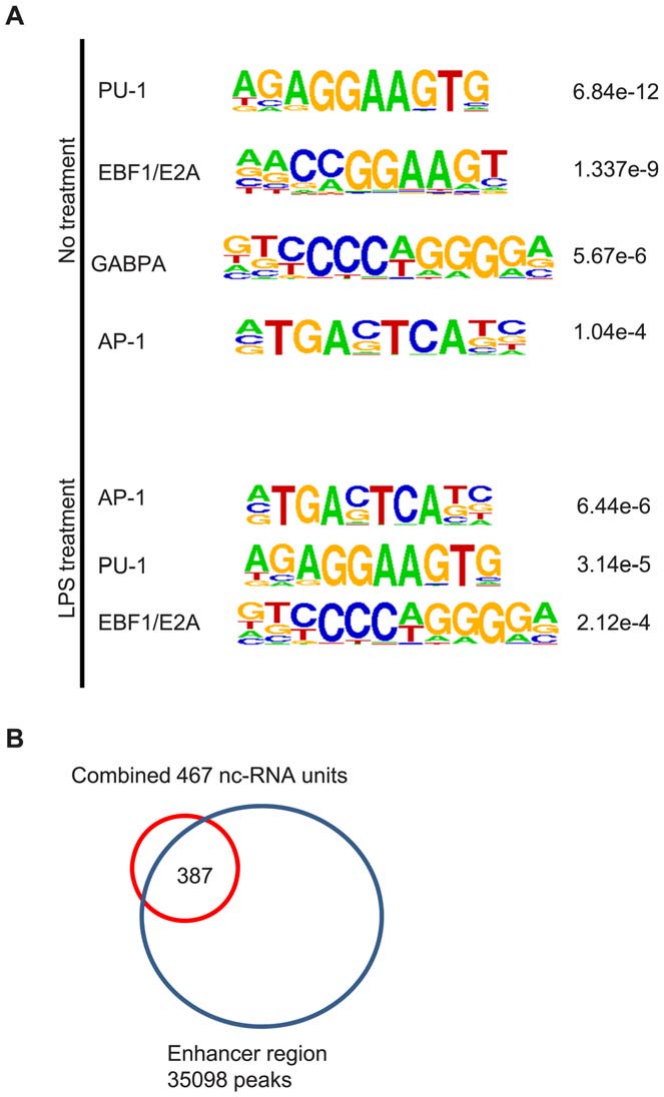
Association of lincRNAs with enhancer markers. A: motifs enriched in the promoter regions of lincRNA that are defined as sequences within the H3K4Me3 clusters of the lincRNAs. B: Venn diagram overlap between lincRNA and enhancer regions that are labeled by CHIP-Seq signatures of PU.1 peaks and H3K4Me1 peaks.

We next searched for the enriched motifs existing in the promoter regions that are labeled by H3K4Me3 using the HOMER software [25]. Impressively, PU.1 motif stands out as the most enriched motif under no treatment condition, and is ranked 2^nd^ under the LPS treatment condition (Fig. 6A). PU.1 is an Ets family transcription factor required for the generation of common lymphoid progenitor (CLP) and granulocyte-macrophage progenitor (GMP) cells in the hematopoietic lineage system [19], [26]. CLP ultimately gives rise to B cells and GMP to macrophages. AP-1 is another motif with a significant *P-value*s and it has been shown to be required for macrophage development and function [27]. Given the observation that PU.1 motif is highly enriched, we asked the question whether the putative lincRNA sites overlap with so-called “enhancers”, as reported by De Santa et al. [28]. We collected the “enhancer” signatures that are represented by the overlapping PU.1 CHIP-Seq peaks and H3K4Me1 peaks [29] in enhancer regions. The overlap result shows that among the 467 putative lincRNAs combined from LPS treatment and no treatment conditions, over 387 (83%, significant with a binomial distribution P-value<2.624170e-141) of the putative lincRNAs have enhancer signatures residing within (Fig. 6B).

**Figure 7:**
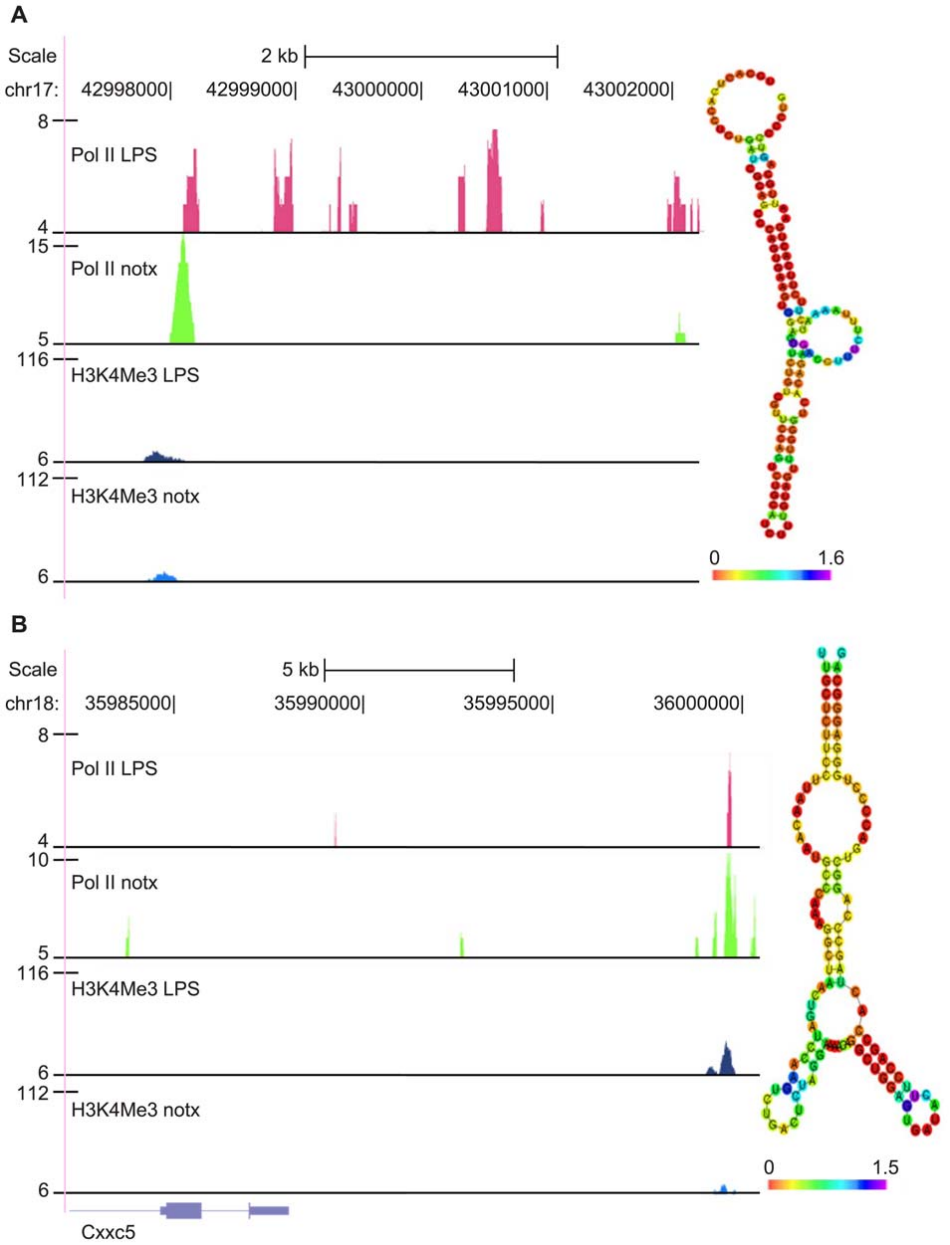
UCSC genome browser (mm8) snapshots of lincRNA examples (left), as well as their conserved, thermodynamically stable 2^nd^ structures predicted by RNAz (right). A: lincRNA located 5′ distal side of Pla2g7 (phospholipase A2, group VII), whose Pol II tags are sensitively stimulated by LPS. B: lincRNA located 3′ distal side of Cxxc5 (CXXC-type zinc finger protein 5), whose Pol II tags are sensitively repressed by LPS.

Non-coding RNAs may harbor conserved secondary structures that can be processed into novel small RNAs, which function as binding partners for transcription factors [1], [30]. Identification of functional lincRNA secondary structures can facilitate discoveries in these regards. Therefore, we checked the existence of conserved secondary structure existing in the transcripts of the putative lincRNAs, using the RNAz prediction algorithm. We found prevalent, conserved secondary structures in the H3K4Me3-free regions of the putative lincRNA transcripts. With a stringent threshold P-value of 0.9, there are 64 windows (120 bp, slide size = 40 bp) that clustered into 53 loci in the LPS treatment group, and 36 windows that clustered in 33 loci in the untreated group. We exemplify two interesting putative lincRNAs as the following: (1) putative lincRNA located 5′ distal side of *Pla2g7*. This lincRNA is sensitively stimulated by LPS treatment, and has high pair-wise identity (90.14) and low free energy of the thermodynamic −38.03 kcal/mol at position 43006516– 43006636 (Fig. 7A). Pla2g7 mRNA expression was shown to increase in blood monocytes and plaque macrophages that were under inflammatory stress [31]. Another example is the putative lincRNA located at 3′ distal side of *Cxxc5*. This lincRNA appears to be negatively affected by LPS treatment and has a highly conserved secondary structure at position Chr18: 35994018–35994129 (Fig. 7B). Cxxc5 is reported as the positive regulator of IKb-kinase, which reciprocally turns on the NFkB pathway [32]. It will be interesting to test experimentally whether this lincRNA repress the expression of the nearby *Cxxc5* gene.

## Discussion

ChIP-Seq with signatures such as histone modifications and protein binding distributions has emerged as a new trend to predict critical genomic features. However, most current CHIP-Seq algorithms have been designed for analysis of sharp peaks from the transcription factors. There only exist a very few computational methods, such as SICER, for dealing with diffuse, broad peaks. One might propose that increasing sequencing depth could fill the gaps among diffuse peaks, but we argue this strategy is not practical. First of all, we are not aware of any computational method capable of predicting tag counts that need to be sequenced to reach saturation for diffuse CHIP-Seq signals prior to experiments, second, for lincRNAs that are very long, complete coverage over the entire transcripts seems very difficult for experiments such as Pol II CHIP-Seq, and even if saturation could be achieved so that all lincRNA transcripts have signals, it will be too costly. GCLS has its unique merit in this regard, and it complements the other computational methods. Unlikely SICER that utilizes an “island approach” to merge nearby clusters, GCLS relies on the global pattern that some neighboring peaks (Type 1 peaks) are highly correlated with the interpeak distance, whereas others (Type 2 peaks) are not. Type 1 peaks reflect that a limited amount of enzymes compete for specific genomic loci, assuming the total loading capacity of the enzymes within a particular actively transcribed locus is relatively constant. Due to the stochastic nature, the more likely one sees some enzymes aggregating closely as one single, wide peak, the farther apart one would expect to see the other competing enzymes near that particular locus. These peaks should therefore be merged to create a single transcription unit. Type 2 peaks appear on the boundaries of transcription units where there is a larger separation between successive signals. These peaks do not need to be merged, as that would be more likely to merge different transcription units. The simulation results based on the diffusion-reaction mechanism further confirmed the plausibility of our reasoning. Based on this initial observation, GCLS algorithm aims to reconstruct the most correct transcription units from these peak components.

GCLS proved effective through validation at several levels. It displayed superior performance to SICER, which relies on a rigid cut-off threshold of gap distance, on the known protein coding genes. GCLS method has the least discrepancy between the total number of Refseq regions and the actual Refseq gene counts, compared to the results of SICER. Additionally, The flexibility of allowing varying inter-peak gaps in GCLS enables the best coverage in the protein coding regions of RefSeq genes, yet much lower noise in the 5′ upstream of TSSs and 3′ downstream of TESs. This advantage is further manifested by the larger number of putative lincRNAs and larger length variation found by GCLS, when compared to SICER. The fact that 8 out 11 putative lincRNAs identified by GCLS were responsive to LPS from QPCR results increases our confidence in GCLS.

Additionally, we carried out extensive computational characterization of GCLS with its application to lincRNA. GO analysis and KEGG analysis on the nearest RefSeq genes associated with these putative lincRNAs show that they are associated with fundamental cellular processes without LPS treatment, but upon LPS treatment, they are shifted to be associated with immune cell specific processes. Also consistent with other studies, the predicted exons of the putative lincRNAs have modest conservation over introns of protein coding genes, but appear to be much less conserved than the exons of protein coding genes [3], [4], [24], [33]. Promoter analysis of the putative lincRNAs shows that they are conserved and enriched with motifs such as PU.1 and AP.1, which are important in determining macrophage lineage. Over 80% of the identified putative lincRNAs overlap with distal enhancers that are characterized by CHIP-Seq signatures of PU.1 and H3K4Me1, surprisingly similar to the observation by others that 84.4% of the enhancer-type Pol II clusters are associated with PU.1 binding [28]. These results strongly suggest that lincRNAs are functionally important. One possible mechanism is that lincRNAs serve as “anchoring” points through transcription factors such as PU.1 to initiate chromosome remodeling and local epigenetic regulation such as H3K4Me1, which in turn might regulate nearby gene activation [1], [25], [28], [29]. Although it is beyond the scope of this paper, some lincRNAs of interest are subject to experimental tests in the collaboration.

The number of putative lincRNAs identified in our report is comparable to those from Guttman et al. [24], but only a relatively small portion of lincRNAs in macrophages overlap with those previously reported in [3]. This result was not unexpected, as Guttman et al. also pointed out that the lincRNAs are likely to be more tissue specific than the protein coding genes, evident by a relatively small number of overlapping lincRNAs between two mouse cell types studied in parallel under the same RNA-Seq platform [24]. Though beyond the scope of this paper, it will be interesting to evaluate lincRNA discovery by different technologies, such as CHIP-Seq vs. RNA-Seq. We envision that Pol II CHIP-Seq, RNA-Seq, and histone chromatin map methods can complement each other to convey different facets of lincRNAs, and further studies on the same cell type are needed for more comprehensive examination of these experimental methods, as well as computational methods based on them.

## Methods

### Generation of ChIP-Seq Data

ChIP-Seq experiments were performed on Genome Analyzers I and II according to protocols of the manufacturer (Illumina). The first 23 bps for each sequence tags were used for alignment to mouse mm8 assembly using ELAND allowing up to 2 mismatches. Only tags that were uniquely mapped to the genome were considered for peak analysis. Two lanes of Pol II ChIP-Seq data were pulled together, under both the Kdo2 lipid A treatment (a purified LPS lipid) for one hour or no treatment conditions, so were the H3K4Me3 ChIP-Seq experiments. One lane of input genomic DNAs (without antibody) was also sequenced as the control sample.

### Identification of intergeneic Chip-Seq Peaks

The data in this study were obtained from primary macrophage cells under resting and activated conditions [10]. Some raw data were deposited in GEO with the accession number GSE21512. The genomic ChIP-Seq peaks were identified similarly to others using our in-house Perl script [8]. Tags were extended to expected length (150 bp) prior to sequencing. No peak shift was adjusted like in CHIP-Seq experiments of transcription factor, due to the diffuse nature of the Pol II CHIP-Seq peaks. Duplicated tags were discarded to avoid PCR artifacts. Peak height was defined as the summit of a continuous region of overlapping tags that are stacked together. Putative peak regions were selected using two-step filtering. First, a null Poisson model with the same number of total mappable tags was used to compute the threshold of peaks height that has FDR<0.001. Peaks that had heights larger than this threshold were selected. A second selection was applied against the input lane (with no antibody) so that the peaks in the experimental lanes must have at least 3X-normalized height as the peaks at the same loci in the input lane. For each condition, a “bed” file was created to visualize the peaks as “custom track” in UCSC genome browser.

To identify intergenic ncRNAs, peaks that overlapped with all RefSeq genes and known pre-microRNA regions were excluded. The mm8 homologs for human (hg18), dog (canFam2), and rat (Rn4) protein coding genes also were excluded, using the liftOver tool in UCSC Genome Browser, similar to [3].

### Classification and Merging Correlated Pol II Peaks into Non-coding RNA Units

We implemented an algorithm to reconstruct the most correct full transcription unit from these peak components. We first separated out the Type 1 peaks from the remaining peaks by determining the best linear separator that went through the point of minimum density between the two groups. The Type 1 peaks are located upper-left to the linear seperator. The density in this case is determined by the number of data points in a region where each data point is plotted based on the log of the base pair width of the tag versus the log of separation in base pairs of successive tags.

Assume 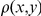 describes the normalized two-dimensional density of the distribution, and 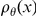 represents the projection of this density distribution onto a line that forms an angle of 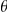 with the *x*-axis. Also, let 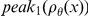 and 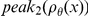 represent the maximum value of the two peaks of the density function and 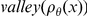 represent the minimum value between those two maxima. Similarly, let 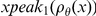, 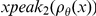, and 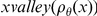 represent the x value at which the respective maxima or minima are reached. Then the slope of the best linear separator can be found tangential to the angle that leads to the largest peak-to-valley difference, which can also be represented in the following formulation:
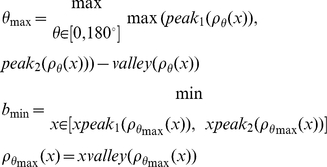



Using these parameters, the linear separator can be computed as the array 
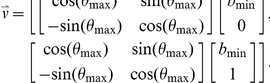


 Once we determined the separator, we merged the Type 1 peaks with their nearest neighbors that were also classified as Type 1 peaks in a given iteration and were located upper-left to the separation line. After merging, we replotted the peak data and found there may still be some Type 1 peaks and so we continued to iterate on merging Type 1 peaks together until the final Pol II units were constructed. As shown in the pseudocode, the stopping criteria for the algorithm were selected to be when the total percentage of peaks merged at each iteration was large enough (greater than 30% of the total peaks) and the total number of merged peaks was large enough (greater than five). We clustered the H3K4Me3 units in the same way. Finally, we ensured that all the lincRNA units have the overlapping signatures of Pol II units and H3K4Me3 units. The pseudo-code for forming the clusters is presented in the Text S2, and the Matlab code for global clustering is available upon request.

### Simulation of the two-cluster pattern as a reation-diffusion phenomena

To verify the distribution observations, we constructed a simplified simulation of the Pol II signals modeled over time assuming one-dimensional reaction-diffusion with the Pol II enzyme (signal) and the binding sites. Initially, the distribution of signal was assumed to be uniform and represented by an array of floating point values where each array element represented the signal over a fixed number of base pairs (*nbp*). In each time step, several computations were carried out. First, a number of potential binding locations (*nb*) were randomly selected. If a binding activity occurred because a gene was present at the location, the signal was amplified in that location by a specified factor (*fa*). Directionality of the signal amplification was randomly selected and occurred over a fixed length of the array (*fl*). Noise was also simulated by allowing a fixed percentage (*fp*) of binding activities not falling on a gene to also result in a reaction event. The resulting signal expression was renormalized so that the total signal added up to unity. The diffusion of the signal followed Fick's second law with a selected diffusion coefficient (*D*). Finally, the simulated Pol II expression levels are found by taking regions of the signal array that are above the mean value of the signal array plus a factor (*fstd*) times the standard deviation of the signal. Figure S3 shows a resulting distribution after a time evolution of these signal peaks that reveals a similar pattern as to what was found in the observed data. The first 300 Mbps of Chromosome 1 were selected for the representative gene data for this simulation. nbp = 100, nb = 1000, fa = 1.05, fl = 7, fp = 0.1, D = 1, fstd = 0.6.

### Comparison to SICER Results

The software SICER was downloaded from http://home.gwu.edu/~wpeng/Software.htm. We also obtained the necessary Python compiler, numpy and scipy packages for running SICER. We used the whole genome Pol II peaks information in experimental lanes against those in the input lane, and chose the following parameters: window size 200 bp, gap size 600/1200/ 2200 bp, and conventionally accepted FDR 0.001. The gap sizes are designated as the multiplication of the window size, and the three gap sizes for SICER are intended to cover a reasonably wide range, based on the observation that average gap size on the RefSeq genes is about 1200 bp. We filtered out the island clusters that overlapped with mouse RefSeq genes, and mouse homologs of human, rat, and dog genes. To estimate the cut-off value of islands' span, we also ran SICER to obtain the intergenic islands from the input lane information alone, with the expected background island number equal to those in experimental data. We then chose the 95% upper quintile of the background island span (3 k bp) as the minimum span for positive Pol II islands. We did similar clustering with the H3K4Me3 data using SICER. We used the whole mouse genome microarray gene expression as testing examples to compare the performance of GCLS vs. SICER, the results were shown in a Receiver Operator Curve (ROC) plot. All data are MIAME compliant.

We could not get access to the code of Guttman et al., and relied on their published coordinates of ∼1600 lincRNAs for comparison. These lincRNAs were found in mouse embryonic stem cells (ESCs), mouse embryonic fibroblasts (MEF), mouse lung fibroblasts (MLF) and neural precursor cells (NPC), rather than macrophages [3].

### Validatition of lincRNAs by QPCR

We randomly selected 11 regions (Table 1), to validate whether they do harbor lincRNAs. Within these regions, 6 have increased Pol II tag counts by the LPS treatment, and 4 have reduced Pol II tag counts by the LPS treatment. LincRNAs were reported to have features of exons [3]. In order to test whether the lincRNAs are truly expressed, we decided to conduct QPCRs in the exons, but not the introns of lincRNAs. We used GENSCAN (http://genes.mit.edu/GENSCAN.html) as a fast approach to predict the exons computationally (P>0.10) [34]. We tested the accuracy of GENESCAN with the Refseq genes on the positive strand of Chromosome 1, and obtained near 80% coverage overall (data not shown). In most cases, we designed 2–3 pairs of primers spanning the selected exons (100–200 bp) in each region of a lincRNA (primer sequences and the predicted exon sequences are in the Text S1). We then did QPCR validation on RNAs extracted from both the bone marrow derived primary macrophages under no treatment, or LPS treatment for 3 hours.

### Computational Characterization of lincNAs

We predicted the functions of the lincRNA by assigning its membership to the nearest RefSeq gene. We then used Database for Annotation, Visualization and Integrated Discovery (DAVID) to perform GO analysis and KEGG pathway analysis to find the terms that are significant [35].

To show the enrichment of H3K4Me3 signals on promoter regions of the lincRNA, we first determined their +/− strand orientation by comparing the mass center of H3K4Me3 on each Pol II transcription unit, then aligned all sequences [−3 k, 1 k] bp around the putative transcription starting sites (TSS) that are determined by H3K4Me3 termination position. We counted the tag frequency in this interval. We also used this interval to find the positional phastCons17way conservation scores of the lincRNAs. We averaged the phastCon scores by the total sequence counts.

To examine the motifs of promoter regions, we took the regions of the lincRNAs that have H3K4Me3 signatures and subdivided them into 200 bp segments. These segments were then subject to both known motif and *de novo* motif analysis by the HOMER software [25]. The default parameters were chosen for the motif analysis (motif length = 10 bp, background sequences = 50,000). To find the conservation in the predicted exons of the lincRNA, we obtained the phastCons17way conservation scores from the UCSC table browser. For comparisons, we also downloaded the coordinates of all the exons and introns of RefSeq genes, wrote a Perl script that extracted the phastCon scores in these regions, and calculated the accumulative distribution of phastCon scores.

To find the secondary structures of the lincRNAs, we first obtained the putative “transcription regions” from the Pol II units that are free of H3K4Me3 markers. We downloaded RNAz (http://www.tbi.univie.ac.at/~wash/RNAz/), a program that predicts structurally conserved and thermodynamically stable RNA secondary structures in multiple sequence alignments [36]. We used the multiple alignment files (MAF) of human, rat, dog aligned to the mouse genome downloaded from the UCSC genome browser. The MAF file was initially filtered before running RNAz (P-value>0.9). The output of RNAz was then clustered over the windows that are partially overlapped.

### Detection of lincRNAs Regulated by LPS Treatment

Given that the Pol II tag counts difference between the lincRNA under LPS treatment and no treatment condition, we first carried out normalization before performing the statistical tests. We combined the intergenic regions that have Pol II peaks in LPS treatment and/or no treatment, and extracted the tags within, and then determined a linear regression line for normalization. Next we assumed a binomial distribution bin(n,p) of the Pol II tags in the same lincRNA regions from LPS treatment vs. no treatment, where n is the normalized tag sum from LPS treatment and no treatment, and p = 0.5, similar to [37]. We chose the Bonferroni corrected P-value 0.05 for the significance threshold.

## Supporting Information

Figure S1 **Density heat-map plot of the emerging genome-wide patterns of Pol II CHIP-seq peaks in RefSeq genes and intergenic regions.** Data are displayed as log10 transformation of the width of peaks vs. log10 transformation of the distance between two successive peak centers. A: pattern of Pol II CHIP-Seq peaks in RefSeq genes. B: pattern of Pol II CHIP-Seq peaks in intergenic regions.
(TIF)

Figure S2 **Evidence of two clusters with K-mean clustering methods (K = 2).** The ‘cosine’ metric was used for clustering. Data are displayed as log10 transformation of the width of peaks vs. log10 transformation of the distance between two successive peak centers. Red data points (type 1) denote peaks that appear to be linearly correlated between peak width and inter-peak distance. Blue data points (type II) denote peaks that lack the linear correlation between peak width and inter-peak distance.
(TIF)

Figure S3 **Reaction-diffusion simulation creates similar two-cluster CHIP-Seq pattern.** The first 300 Mbps of Chromosome 1 were selected for the representative gene data for this simulation. We used the following parameters (described in Methods) run over 80 time-steps: *nbp* = 100, *nb* = 1000, *fa* = 1.05, *fl* = 7, *fp* = 0.1, *D* = 1, *fstd* = 0.6. Data are displayed as log10 transformation of the width of peaks vs. log10 transformation of the distance between two successive peak centers. The resulting distribution appears similar to the observed distribution in the data (Figure 1).
(TIF)

Figure S4 **The RefSeq genes and regions in GCLS overlap those in SICER.** SICER is parameterized over 3 different gap distances: 600 bp, 1200 bp and 2200 bp. A region is defined as the maximum contig of several overlapping genes if there are any, or the locus of one single gene if there are no other overlapping genes.
(TIF)

Figure S5 **The Receiver Operator Curve (ROC) plot based on the binary classification of known genes identified by Pol II vs. measured by microarray gene expression.** GCLS and SICER can extract clusters that overlap with known genes with very similar trends of true positive rate vs. false positive rate. To make binary classification, a gene is classified as “truly expressed” when a cluster overlaps the transcription region of that gene; otherwise, it is classified as “falsely expressed”. To measure the two algorithms, the log2 transformation of the microarray intensities of genes is further normalized to represent the probability of gene expression, ranging on [0, 1].
(TIF)

Figure S6 **PCR validation of lincRNAs in** **Table 1** **, using bone marrow derived macrophage primary cells under control or LPS treatment.** The notation “c2a” denotes “exon a” from the lincRNA in Table 1 that is located on the reverse (or complementary) strand of Chromosome 2. “5b” denotes “exon b” from the lincRNA in Table 1 that is located on the forward strand of Chromosome 5, and so on. The predicted effect of LPS, based on the statistical analysis of Pol II tag counts, is listed under each lincRNA. A region on chrosome 17 (17 d) was used as the negative control. All expression levels are normalized by GAPDH. The exon sequences and primer sets are listed in the Text S1.
(TIF)

Table S1 **The coordinates of lincRNAs under LPS vs. no treatment conditions predicted by GCLS.**
(XLS)

Table S2 **lincRNA overlap among three different computational methods: GCLS, SICER600 and Guttman's method in** [**3**] **.**
(XLS)

Text S1 **Exon sequences that are predicted by GENSCAN from the 11 regions of lincRNAs as shown in** **Table 1** **, as well as primer sets from these exons that were used to validate the effects of LPS treatment.** The strand information and chromosome coordinates are shown in the header of each sequence. The specific primer set is listed right before each exon The predicted exon sequences are in bold and located between two arrow signs. Other regions irrelevant to the exons are omitted. The indices in front of each line in the sequence are relative to the start position of each range.
(DOC)

Text S2 **The pseudo code to separate the clusters.**
(DOC)
