# Supplementary figures and images for: A Global Clustering Algorithm to Identify Long Intergenic Non-Coding RNA - with Applications in Mouse Macrophages
Source: PLoS One. 2011 Sep 30;6(9):e24051. doi: 10.1371/journal.pone.0024051 (PMC3184070; doi:10.1371/journal.pone.0024051)

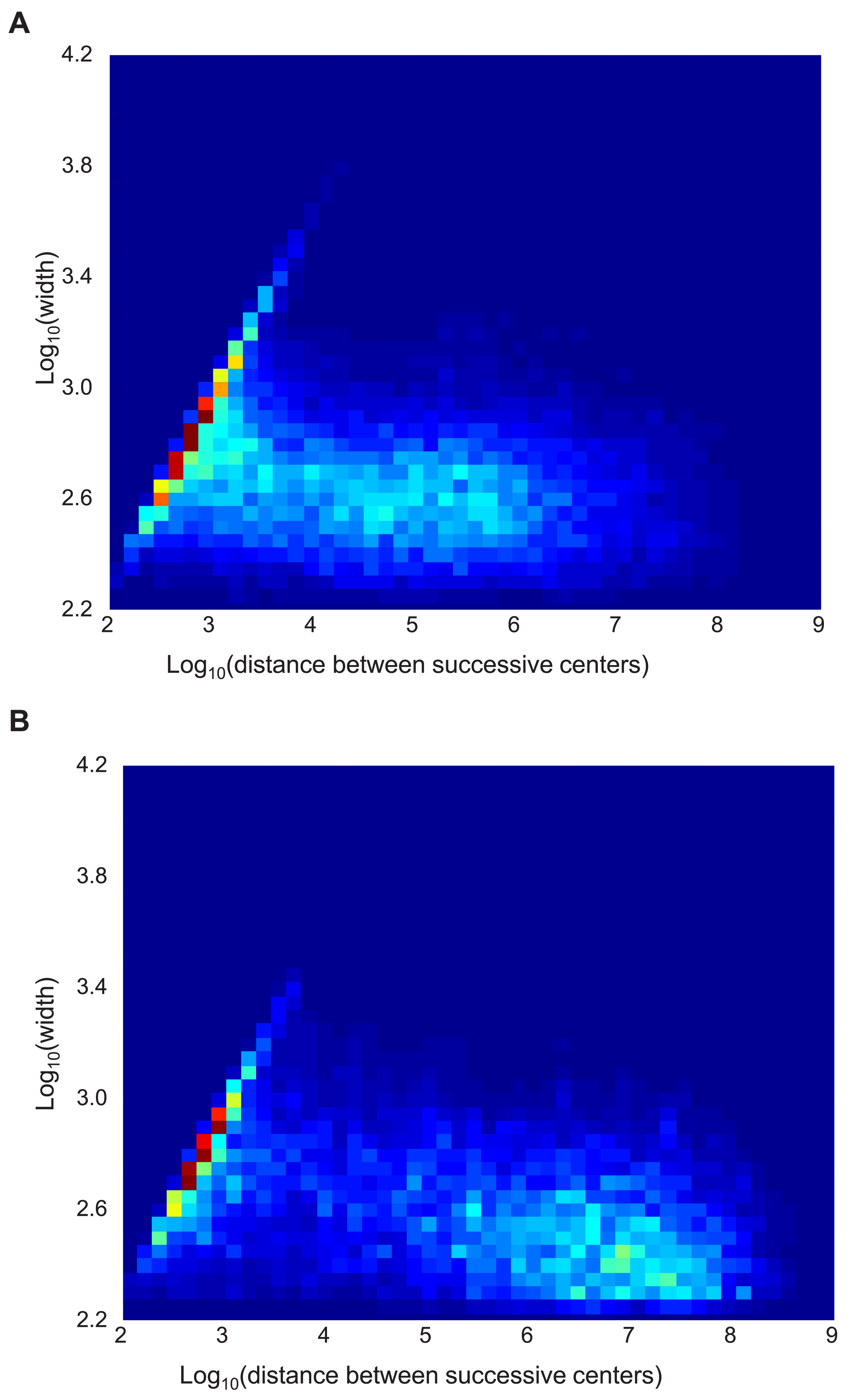

Supplement: Figure S1 — Density heat-map plot of the emerging genome-wide patterns of Pol II CHIP-seq peaks in RefSeq genes and intergenic regions. Data are displayed as log10 transformation of the width of peaks vs. log10 transformation of the distance between two successive peak centers. A: pattern of Pol II CHIP-Seq peaks in RefSeq genes. B: pattern of Pol II CHIP-Seq peaks in intergenic regions. (TIF) [file pone.0024051.s001.tif]

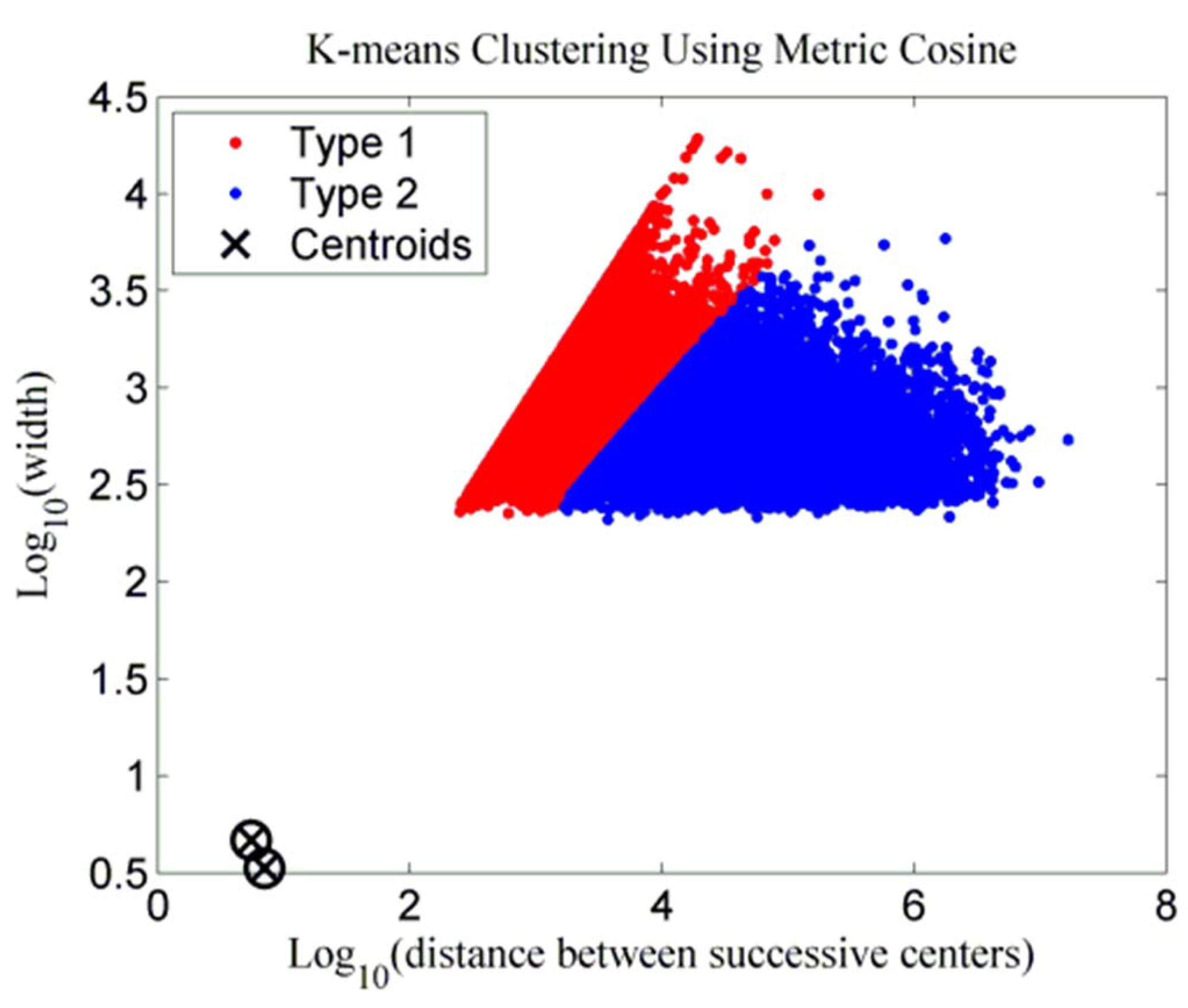

Supplement: Figure S2 — Evidence of two clusters with K-mean clustering methods (K = 2). The ‘cosine’ metric was used for clustering. Data are displayed as log10 transformation of the width of peaks vs. log10 transformation of the distance between two successive peak centers. Red data points (type 1) denote peaks that appear to be linearly correlated between peak width and inter-peak distance. Blue data points (type II) denote peaks that lack the linear correlation between peak width and inter-peak distance. (TIF) [file pone.0024051.s002.tif]

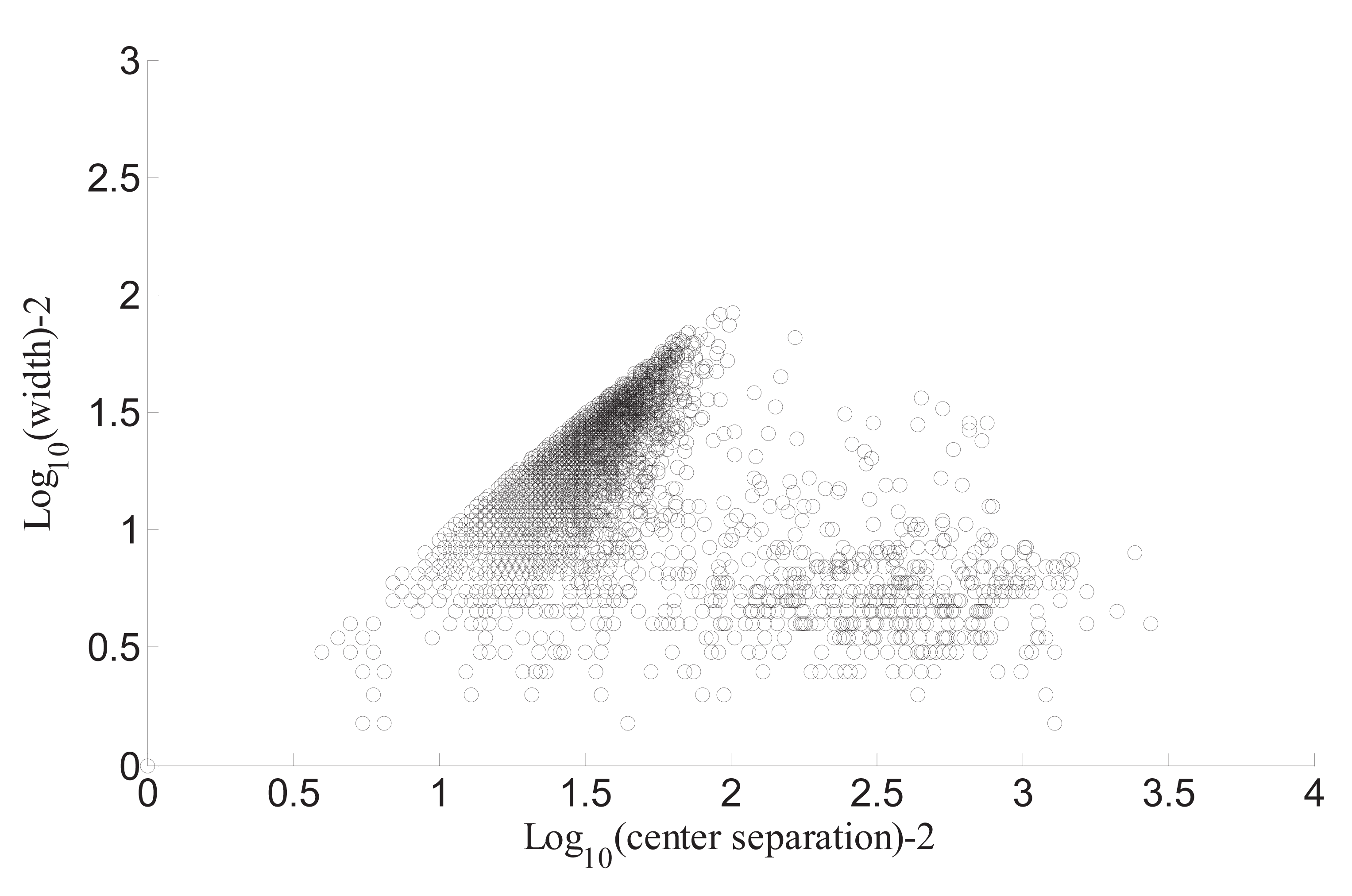

Supplement: Figure S3 — Reaction-diffusion simulation creates similar two-cluster CHIP-Seq pattern. The first 300 Mbps of Chromosome 1 were selected for the representative gene data for this simulation. We used the following parameters (described in Methods) run over 80 time-steps: nbp = 100, nb = 1000, fa = 1.05, fl = 7, fp = 0.1, D = 1, fstd = 0.6. Data are displayed as log10 transformation of the width of peaks vs. log10 transformation of the distance between two successive peak centers. The resulting distribution appears similar to the observed distribution in the data (Figure 1). (TIF) [file pone.0024051.s003.tif]

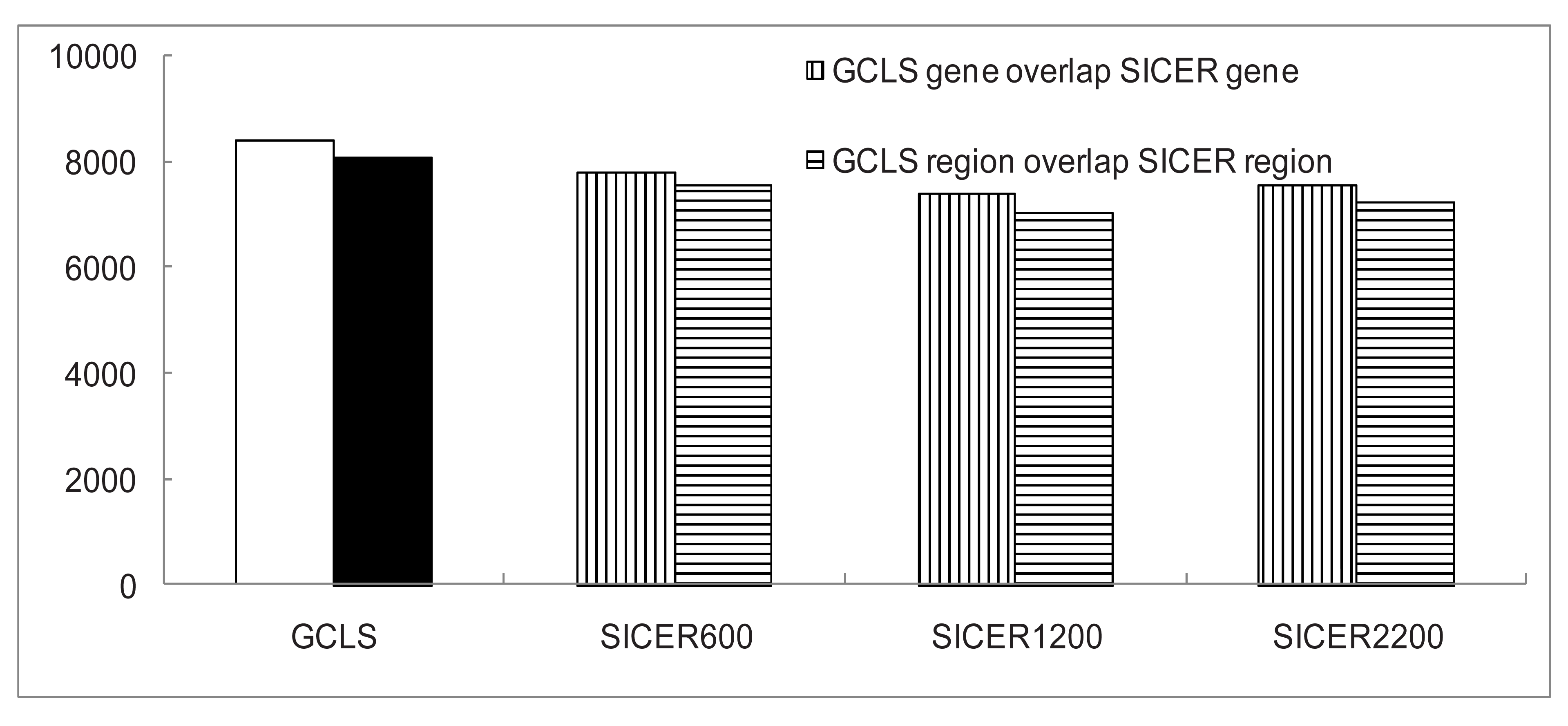

Supplement: Figure S4 — The RefSeq genes and regions in GCLS overlap those in SICER. SICER is parameterized over 3 different gap distances: 600 bp, 1200 bp and 2200 bp. A region is defined as the maximum contig of several overlapping genes if there are any, or the locus of one single gene if there are no other overlapping genes. (TIF) [file pone.0024051.s004.tif]

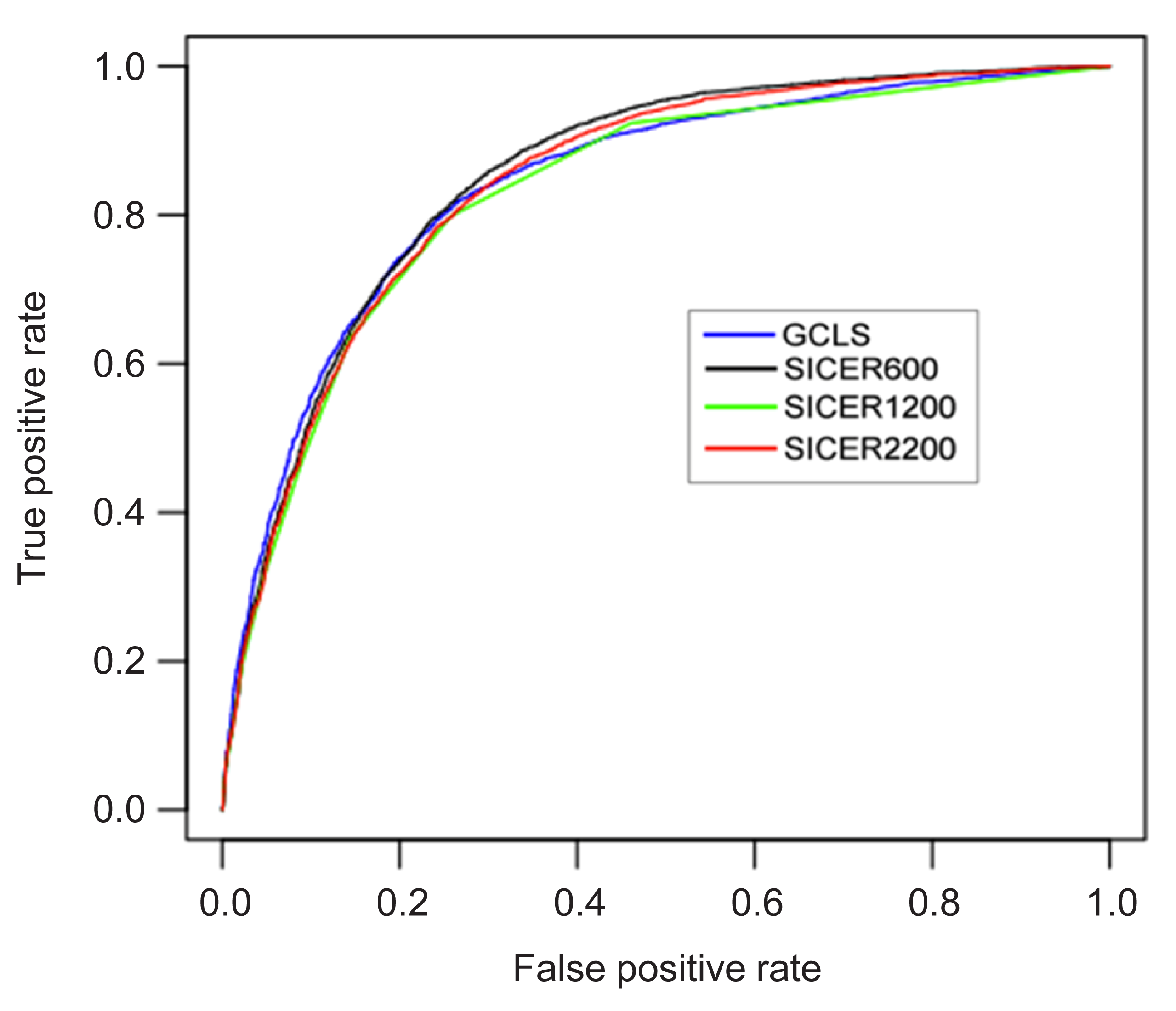

Supplement: Figure S5 — The Receiver Operator Curve (ROC) plot based on the binary classification of known genes identified by Pol II vs. measured by microarray gene expression. GCLS and SICER can extract clusters that overlap with known genes with very similar trends of true positive rate vs. false positive rate. To make binary classification, a gene is classified as “truly expressed” when a cluster overlaps the transcription region of that gene; otherwise, it is classified as “falsely expressed”. To measure the two algorithms, the log2 transformation of the microarray intensities of genes is further normalized to represent the probability of gene expression, ranging on [0, 1]. (TIF) [file pone.0024051.s005.tif]

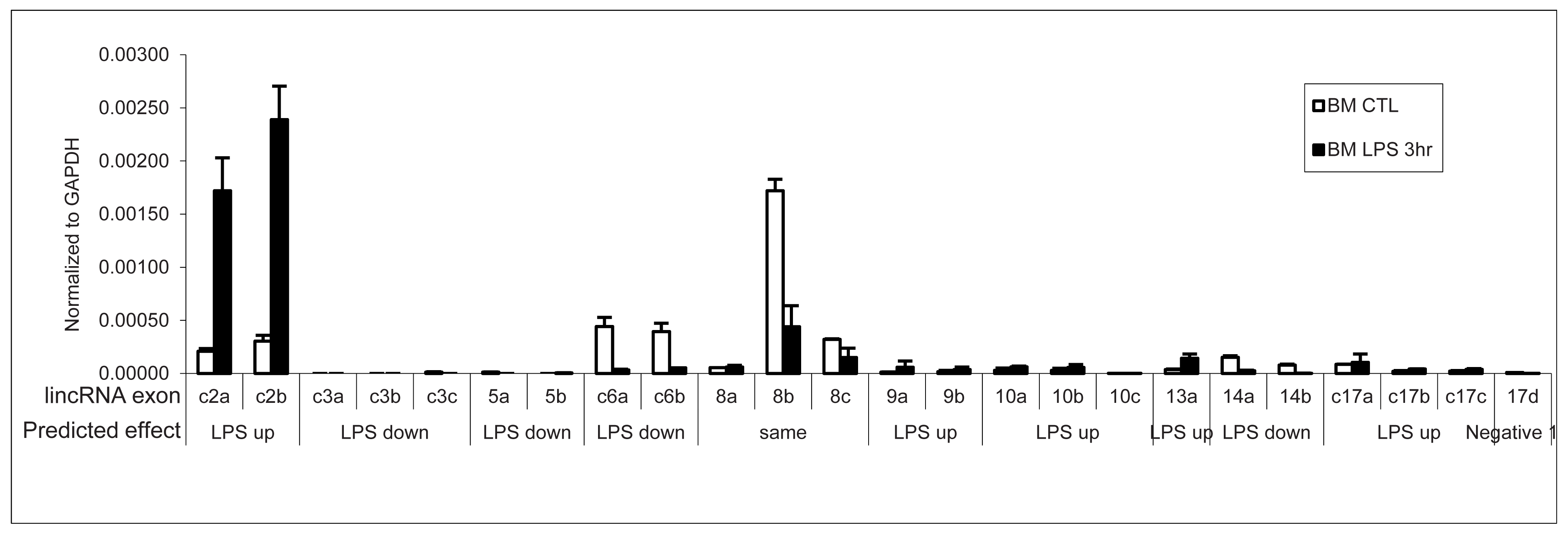

Supplement: Figure S6 — PCR validation of lincRNAs in Table 1 , using bone marrow derived macrophage primary cells under control or LPS treatment. The notation “c2a” denotes “exon a” from the lincRNA in Table 1 that is located on the reverse (or complementary) strand of Chromosome 2. “5b” denotes “exon b” from the lincRNA in Table 1 that is located on the forward strand of Chromosome 5, and so on. The predicted effect of LPS, based on the statistical analysis of Pol II tag counts, is listed under each lincRNA. A region on chrosome 17 (17 d) was used as the negative control. All expression levels are normalized by GAPDH. The exon sequences and primer sets are listed in the Text S1. (TIF) [file pone.0024051.s006.tif]
