# Supplementary material for: A Global Clustering Algorithm to Identify Long Intergenic Non-Coding RNA - with Applications in Mouse Macrophages
Source: PLoS One. 2011 Sep 30;6(9):e24051. doi: 10.1371/journal.pone.0024051 (PMC3184070; doi:10.1371/journal.pone.0024051)
Supplement: Text S1 — Exon sequences that are predicted by GENSCAN from the 11 regions of lincRNAs as shown in Table 1 , as well as primer sets from these exons that were used to validate the effects of LPS treatment. The strand information and chromosome coordinates are shown in the header of each sequence. The specific primer set is listed right before each exon The predicted exon sequences are in bold and located between two arrow signs. Other regions irrelevant to the exons are omitted. The indices in front of each line in the sequence are relative to the start position of each range. (DOC) [file pone.0024051.s009.doc]

**Supplementary Material:**

>mm8_gold_chr2.1 range=chr2:30734001-30755000 5'pad=0 3'pad=0 strand=- repeatMasking=N

.....

| cCh2aF | CCCAGTAGCGTCGTTGATGA |
| --- | --- |
| cCh2aR | CTTGTCTGGGCTTGGGAGAA |

2350 ACTGTAGATGACTCGCC->**GTATTACCAAACCGGGTCATCGCAGCAACTACT**

**2400 TCACGACTCAACCTCAAAATACGTTCCGAAGATCGTGAAGGTTCCGAAGA**

**2450 GGGTTCGGGTCTGTTCGAGGATTGTGGACGAGGGTCCACCGGTAGTCCCG**

**2500 ATTATGGAAAGACAAATACTTGTCCACTGTCGTG**<-TCACAGGGACTTGTAA

.....

| cCh2bF | CCTGTACCAGATGCCTCCTT |
| --- | --- |
| cCh2bR | TGAGGAAAGTGGACCCTGTT |

13250 GTGTCCTACATCTGTATCGTGCTCGAGACGATTGACC->**CCGAGGACATGGT**

**13300 CTACGGAGGAAGATGAAGTAACTCTTTGTCCCAGGTGAAAGGAGTAGGAC**

**13350 CTCTCTGGT**<-CTCACGAGACGACGGGACTAATCAGTGGGGTCGACCGTGGT

>mm8_gold_chr3.1 range=chr3:84730001-84735000 5'pad=0 3'pad=0 strand=- repeatMasking=N

.....

| cCh3aF | CTGCCCTGTTCTCGTTTCTA |
| --- | --- |
| cCh3aR | GTTTCTTTCCCGGGTGACAT |

800 CTCGACCGTAT->**ACACCACAACTGGTCTCTGAGGACTGTCCGACCAAGGAG**

**850 GACCCGGGGACGGGACAAGAGCAAAGATACCAAACCCAGTTACGTCTGGA**

**900 AGACTCCTTCCGATCTTCTGTTTCATCTCAGTGCACTTTTGTCAAGAACA**

**950 CGAGCTCTGTCCTCCGTACCGCACGGTGGATACAGTGGGCCCTTTCTTTG**

**1000 GGTACACAGGTCTGGTCT**<-CTGTCTCCGTACTCTTCGCAGTCAGACCCCAG

.....

| cCh3bF | TGAACTACTGGGGTGCTCTC |
| --- | --- |
| cCh3bR | GCTTCCTCTTTAAAGCAAACT |

1050 ACATAACACCGAAGGAACCGTTCCGTATCGTCT**ACTTGATGACCCCACGA**

**1100 GAGACCACTCCCACGAAACCCCCACGATACTCGATACCGTTGTCAAAACG**

**1150 AAGTAGACAATGGTCTATTCAAACGAAATTTCTCCTTCGTTCAGACCTC**<-T

.....

| cCh3cF | GGCTTGACAGTGCAGAGCAT |
| --- | --- |
| cCh3cR | CCTGGGTGAGTCAGTACGAA |

1900 GATCTATCAAGACCTGAACTTAGAACTAGGGTTTTCCT->**GGGACCGAACTG**

**1950 TCACGTCTCGTAAGAAAGACGTTCACCGGTCGGACCCCGGAGATGACATG**

**2000 GTCGCAAAGCATGACTGAGTGGGTCCGTCGCGAAACGATCTGTTAAATGA**

**2050 CAAAAAGACAT**<-CGGAAGTTTGAGGTCGATAATTAAAAACTCACACAAATC

>mm8_gold_chr5.1 range=chr5:37283501-37290000 5'pad=0 3'pad=0 strand=+ repeatMasking=N

.....

| Ch5aF | ACTTCCGGTTTTCCTCTGCT |
| --- | --- |
| Ch5aR | CTGTCGGCACTCACGAACT |

6000 GAGCAGGAGCCGA->**GCACTTCCGGTTTTCCTCTGCTGGCAAGGCCCAGAGC**

**6050 ACTGCGCTGGCAGCTGCTCCTGGGAGAGTTCGTGAGTGCCGACAGCCGGA**

**6100 GTCCAGGGGCCTGAGGGAAGCACC**<-AGCACCCAGTGCTGCTCCTAGCTTTT

.....

| Ch5bF | AGACCACAGGCAAGACGAAT |
| --- | --- |
| Ch5bR | GCCCCAGAACTTTCCAAGG |

6350 TCCTAGGTGGGACAGACCCCCCATTTCAGCAAGCTTCAGGTCTCCTAGA->**G**

**6400 CAGACCACAGGCAAGACGAATGTCCTGTTTGTGGACATAAGGGTGTGGAG**

**6450 CCTGCTTTCATTTCCTTGGAAAGTTCTGGGGCCCA**<-GCATGGAAGACTCCA

>mm8_gold_chr6.1 range=chr6:48950001-48964000 5'pad=0 3'pad=0 strand=- repeatMasking=N

**.....**

| cCh6aF | TGGCCCTCATTTGAGCAGGA |
| --- | --- |
| cCh6aR | AAAGGAGGGCTGGAATGGTA |

600 GTCCCACTCCCCCACTGT->**ACGACACGTGTCACCGGGAGTAAACTCGTCCT**

**650 TACGGCAAGTCCACGGACGACACGGAGGGACCACGGTCCGGGTCGACCGA**

**700 GAGATGGTAAGGTCGGGAGGAAACGGTGTAGTCAAACAAGAATTAAGGA**<-A

.....

| cCh6bF | TAGGGAAGAGAGGAGGAAGC |
| --- | --- |
| cCh6bR | TTGGGTGAGGCTCAGGAAGA |

6150 TGTTGTCTGTGTGTCCCTTTTAGACCCCAACCCACGCGACATGACCGAGA

6200 CTATCTCCGATGGGAG->**ATATTGAGTTGTCGTGTAATACATATCTTGTTCT**

**6250 ACAACCGATTAGAGCCAACCTCCGGAGACATCCCTTCTCTCCTCCTTCGA**

**6300 CACCAACTATCAAAGATGTATGTGACCAAAATCGATTTCCTGGATGGTCG**

**6350 GCATTGTGAATATGTCTGGTCTCCTTCCGTAAGGGTAGAAGGACTCGGAG**

**6400 TGGGTTTCTTTCAAGACGGTG**<-AGGGACGCCGACTCCGTCACTTCAGGAA

…

>mm8_gold_chr8.1 range=chr8:87090001-87103500 5'pad=0 3'pad=0 strand=+ repeatMasking=N

| Ch8aF | CTTGGCTGGGTTTTGTTCCT |
| --- | --- |
| Ch8aR | TGCATGTCTCTCCCTCTTCA |

300 AAGTAGCAGTCCAACATGCCCAAGGAGGTTGGCGTGG->CCACAGAGGGCA<-C

350 CCTGCCCC->**TCCTTGGCTGGGTTTTGTTCCTGGCCCCCTACCTGGGCACCA**

**400 GGCCAGGCCCTGAGGCAGGGCCCCGTCCAGGGGAACAATACCACCGTGTT**

**450 CCTGATGGTGGAGGGGAGGCCTTAAGGATGAAGAGGGAGAGACATGCACA**<-

…

| Ch8bF | ACCTGGGGTGTATGAGACCA |
| --- | --- |
| Ch8bR | CATCTTGCCAGTCAGTGCAT |

9950 TAGTTCTGTGGTCAGTAGGTGCTGCGACCT->**GAAGATCCTGGGATCCTTTG**

**10000 GGGATGGCTTGAACGGGAGGGATAACCCAGACCTGGGGTGTATGAGACCA**

**10050 ATCTGTGGTCACCCCAGTTCCATGAGGGAGTATGTTACAAGAATGTACTC**

**10100 TGCCTATGCTGGGATGCACTGACTGGCAAGATGCCAGGGCTTAAGGACGG**

**10150 GCACATATGCGCGNN**<-NNNNNNNNNNNNNNNNNNNNNNNNNNNNNNNNNNN

.....

| Ch8cF | TGAAGGACAGTGCAATCTGG |
| --- | --- |
| Ch8cR | CCTCTTGCTGGTGGCTTTAC |

10650 GTCACTGGGGACACAGGCGTCCC->**CAGCTGCCCTCCGCACCTGGTGAGACC**

**10700 TCCGGAGCTCCGAATGACGTTGTCTGGAGCCAGACGCCCCCTGGTGGTCA**

**10750 TTTCCTGCAATGAAGGACAGTGCAATCTGGGTGCATTTTCCAAAAACCTA**

**10800 CAGACTCTAGGTTTGGGCGTTTTAAGCCTCCTGACTTGCAGAGATTATGA**

**10850 CATGCAGATGGAAAAGCAGTAAAGCCACCAGCAAGAGGTGGCTCTTCGTG**

**10900 TCACCACGATGCCAT**<-GAAGAAACCAGCAGCCTCTCCTGTCCCATTCAAGT

>mm8_gold_chr9.1 range=chr9:119857501-119863000 5'pad=0 3'pad=0 strand=+ repeatMasking=N

| Ch9aF | GGATAGATGGAGAGGATCAAGG |
| --- | --- |
| Ch9aR | CCAGTCATAAAACACACTGGTTG |

1050 AGAA->**AAAGGATAGATGGAGAGGATCAAGGTCCTGGGGCAGGAACCAACCA**

**1100 GTGTGTTTTATGACTGGAGGA**<-GGAGGGTAAGGAGGAGCTGGGGCCTGGGG

.....

| Ch9bF | GCAAGGACAGAGCATCGAG |
| --- | --- |
| Ch9bR | CTCCCTCCCAGATTCCAAAC |

2200 ATAGAGCATCATACTGGGGAGGCAGAGACAGATGAAAGCGGGAAGCA->**GAG**

**2250 GACGAATTCCAGACGCTGGCAAGGACAGAGCATCGAGGGACCAGGCGCCC**

**2300 TCCCGGGTGAGCCCCGGAACCGGCATCCGTTTGGAATCTGGGAGGGAGG**<-C

.....

>mm8_gold_chr10.1 range=chr10:18750001-18758500 5'pad=0 3'pad=0 strand=+ repeatMasking=N

….

| Ch10aF | TCACTTTTAACCAGCTATCAGTCC |
| --- | --- |
| Ch10aR | AACCACCTAACGGCCAGAGT |

2150 CTGTTTCTAAACTCGCAGACAGCCACCAGT->**ATGTCTCAGTTGTCACTTTT**

**2200 AACCAGCTATCAGTCCAGCTTGCTGTTGCTCTTTTATCTGTAAGTGAGGC**

**2250 AGACTCTGGCCGTTAGGTGGTTGATACATTTG**<-ATAATCTTAATTAATTTG

.....

| Ch10bF | TGTGTTCTGCATGTTCCACA |
| --- | --- |
| Ch10bR | CCCACATCAGGGCTAAAGAG |

2800 CAGTGTGGTCCTATAGCAAAACTCAGAACATTGGTGATGGTTC->**GGTGCTT**

**2850 CTCTCCTGTGTTCTGCATGTTCCACAAGGGTAAGGAGCAAGTCCGCTTCT**

**2900 CTTTAGCCCTGATGTGGGACAC**<-ATAAGAGAATCTCAGAAAATGTTGAAGT

.....

| Ch10cF | AGGGACAGCCTACATAAGAGGT |
| --- | --- |
| Ch10cR | TGTATAACCTAGGAAACAGTATCACC |

7150 CAATGT->**CAGGGACAGCCTACATAAGAGGTAGGAGGTGATACTGTTTCCTA**

**7200 GGTTATACACTAAATAG**<-CCAGAACACCAGCAAGTATCATTCCCTCTGCAG

……

>mm8_gold_chr11.1 range=chr11:83349001-83361500 5'pad=0 3'pad=0 strand=+ repeatMasking=N

……

| Ch11aF | CTGTGGAATTTGGCACAATCT |
| --- | --- |
| Ch11aR | TTCCTAGCACTTACTAATACAACATGC |

6550 TGGTGAGCTATAC->**ATTGCTACAATATCTGTGGAATTTGGCACAATCTGTT**

**6600 AAAAATCTGGAAAGAGCATGTTGTATTAGTAAGTGCTAGGAATCT**<-TACTT

……

>mm8_gold_chr13.1 range=chr13:55198801-55202000 5'pad=0 3'pad=0 strand=+ repeatMasking=N

……

| Ch13aF | GGGAGTGGCAAATCCAGATA |
| --- | --- |
| Ch13aR | TGAAGAGCCTTGGGAAAAAG |

1000 GAGCGTTGCTATCAGGGAGTGGCAAATCCAGATAGATTGATGGGCACAGC

1050 GACAGCCGGATCCGGGGCGAACTGGTCTGGACAAGAATTGGCAGTCTCTT

1100 CCCGACTCCGCCCAGTTTGCAAAGCTCACCTTTTTCCCAAGGCTCTTCAT

1150 CCCGCACCAACGTCATGGCCTTGGCTGGGGTGGC->**CGCCGATTGGCCTGTG**

**1200 GCAGCCATGTGACAAAGACGAGTCTTAGCCACCCTCCCCCGAATGCAGTC**

**1250 CCACG**<-TGTGGCCGCTGCGGCCGCGTGGTGTTGTCTGCAGCTTTATCGCGG

>mm8_gold_chr14.1 range=chr14:62032001-62036800 5'pad=0 3'pad=0 strand=+ repeatMasking=N

.....

| Ch14aF | AGGCTGTGGAAACTTCTGGA |
| --- | --- |
| Ch14aR | GAGCTGAACCAGAAAGCAGAA |

1050 ATAGAAA->**ATCTCCTCCACAGGCTCATGGATTTGAAAATGGGCTCTACCTG**

**1100 ACAGCCCTGTTCTCCAAGGCTGTGGAAACTTCTGGACACAGGGCTGTCTA**

**1150 GTCTCATTTGCAGCATGTATAGTTTCTGCTTTCTGGTTCAGCTC**<-GACTTG

.....

| Ch14bF | TCTTCTGTCGAAGATACAGTGAGG |
| --- | --- |
| Ch14bR | ATTGCTGCATCCAAGTTGTG |

3550 ACATGGCTGACACCCAGTCTCCGCCCCTGCCTGTCTCTGTGTCCA->**CTCTT**

**3600 CTGTCGAAGATACAGTGAGGGGTACCTTGTTGAACCCACAACTTGGATGC**

**3650 AGCAATT**<-GGAAGTAGCAAATGGTGGTTTTGAAGTGAACCTTAGGTGGGCT

>mm8_gold_chr17.1 range=chr17:29119501-29124500 5'pad=0 3'pad=0 strand=- repeatMasking=N

……

| cCh17aF | CTCTCAGTCGAGGGTTTCCT |
| --- | --- |
| cCh17aR | GTTATGGGGACCAACCACTC |

400 ACACTGAGAGGGTCTCTGGGAA->**AGGACAAGAAACCAGATCATACGGAGAG**

**450 TCAGCTCCCAAAGGACGTCGACTCCCAGTGGGAGTGGGGGTGTGGGGATA**

**500 GTATGAGAACCAGGATCCTCACCAACCAGGGGTATTGGGGACCCATCGAC**

**550 GGTCCCTACAATTTCCCGGACTCGCTCACAGGTTTACGAAACCATCTGGG**

**600 ATATAAATTTATGTTGGTGTTGCA**<-CATATTAAATGTGTCGGTGCGATACT

……

| cCh17bF | AAAAGGTGACAAGCCTAAGT |
| --- | --- |
| cCh17bR | ACTTGCAGCCAGATGTGCAG |

1850 CCGTATCATCTTTGGGGGT->**TTATTACAGCACTCGACCTTTATCTTTTCCA**

**1900 CTGTTCGGATTCACGGAATCCCGCCCATCCGAAATCCTGAACCTTCCACA**

**1950 GGTGTCGACCTTACGACCGAGGAAGACGTGTAGACCGACGTTCA**<-CACCCT

……

| cCh17cF | AATGGCCCCAGAGAACACAG |
| --- | --- |
| cCh17cR | AGCTAGGCTCTAACTGAAGTG |

2850 GACTCACACAACCACCC->**TGGCCCTCGTCTGGACCTCCTAGACCTTACCGG**

**2900 GGTCTCTTGTGTCGAGTGAAGGAGAACACGGTCCGGGTGGGTCTCCGGGG**

**2950 TCGTTCACGTGAAGTCAATCTCGGATCGA**<-CCCTTTTGGGGACCTCATGTT
