# Supplementary material for: A Global Clustering Algorithm to Identify Long Intergenic Non-Coding RNA - with Applications in Mouse Macrophages
Source: PLoS One. 2011 Sep 30;6(9):e24051. doi: 10.1371/journal.pone.0024051 (PMC3184070; doi:10.1371/journal.pone.0024051)
Supplement: Text S2 — The pseudo code to separate the clusters. (DOC) [file pone.0024051.s010.doc]

previousmerged = largest positive number

totalmerged = previousmerged

percentagemerged = 1

while percentagemerged > 0.3 and totalmerged > 5

totalmerged = 0;

forall j in number of chromosomes

forall i in peaks of chromosome j

X = log10(separation of ith peak from i+1th peak)

Y = log10(width of ith peak);

If (X, Y) is in merge set (using

Merge peak i and i+1

totalmerged = totalmerged + 1

end

end

end

percentagemerged = (previousmerged-totalmerged) / previousmerged

previousmerged = totalmerged;

end
